# CRISPR/Cas9 genome editing of CCR5 combined with C46 HIV-1 fusion inhibitor for cellular resistant to R5 and X4 tropic HIV*-*1

**DOI:** 10.1038/s41598-024-61626-x

**Published:** 2024-05-13

**Authors:** Wannisa Khamaikawin, Chonticha Saisawang, Boonrat Tassaneetrithep, Kanit Bhukhai, Phetcharat Phanthong, Suparerk Borwornpinyo, Angsana Phuphuakrat, Ekawat Pasomsub, Sujittra Chaisavaneeyakorn, Usanarat Anurathapan, Nopporn Apiwattanakul, Suradej Hongeng

**Affiliations:** 1 Faculty of Medicine, King Mongkut’s Institute of Technology Ladkrabang, Bangkok, 10520 Thailand; 2 Center for Advanced Therapeutics, Institute of Molecular Biosciences, Mahidol University, Salaya, Nakhon Pathom, 73170 Thailand; 3 Center of Research Excellence in Immunoregulation, Faculty of Medicine Siriraj Hospital, Mahidol University, Bangkok, 10700 Thailand; 4 Department of Physiology, Faculty of Science, Mahidol University, Bangkok, 10400 Thailand; 5 Department of Anatomy, Faculty of Science, Mahidol University, Bangkok, 10400 Thailand; 6 Department of Biotechnology, Faculty of Science, Mahidol University, Bangkok, 10400 Thailand; 7 Excellent Center for Drug Discovery, Faculty of Science, Mahidol University, Bangkok, 10400 Thailand; 8 Department of Medicine, Faculty of Medicine Ramathibodi Hospital, Mahidol University, Bangkok, 10400 Thailand; 9 Department of Pathology, Faculty of Medicine Ramathibodi Hospital, Mahidol University, Bangkok, 10400 Thailand; 10 Department of Pediatrics, Faculty of Medicine Ramathibodi Hospital, Mahidol University, Bangkok, 10400 Thailand

**Keywords:** CCR5 CRISPR/Cas9, C46 HIV-1 fusion inhibitor, HIV-1 gene therapy, Genetics, Molecular biology, Medical research, Molecular medicine

## Abstract

Hematopoietic stem-cell (HSC) transplantation using a donor with a homozygous mutation in the HIV co-receptor CCR5 (CCR5Δ32/Δ32) holds great promise as a cure for HIV-1. Previously, there were three patients that had been reported to be completely cured from HIV infection by this approach. However, finding a naturally suitable Human Leukocyte Antigen (HLA)-matched homozygous CCR5Δ32 donor is very difficult. The prevalence of this allele is only 1% in the Caucasian population. Therefore, additional sources of CCR5Δ32/Δ32 HSCs are required. The Clustered Regularly Interspaced Short Palindromic Repeats (CRISPR)/CRISPR-associated (Cas) system is one method to mediate CCR5 knockout in HSCs that has been successfully employed as a gene editing tool in clinical trials. Additional anti-HIV-1 strategies are still required for broad-spectrum inhibition of HIV-1 replication. Here in this study, we combined an additional anti-HIV-1 therapy, which is C46, a cell membrane-anchored HIV-1 fusion inhibitor with the CRISPR/Cas9 mediated knockout CCR5. The combined HIV-1 therapeutic genes were investigated for the potential prevention of both CCR5 (R5)- and CXCR4 (X4)-tropic HIV-1 infections in the MT4CCR5 cell line. The combinatorial CRISPR/Cas9 therapies were superior compared to single method therapy for achieving the HIV-1 cure strategy and shows potential for future applications.

## Introduction

Infections with human immunodeficiency virus type 1 (HIV-1) have increased continuously since the first identification of HIV-1 in the early 1980s^1^. The standard treatment for HIV-1 infection is combination antiretroviral therapy with two or more antiretroviral agents, which can efficiently reduce plasma viral loads and maintain CD4 + T-lymphocytes. However, the antiretroviral therapy regimen cannot completely cure HIV-1. The persistence of the viral latent reservoir, especially in the resting memory CD4^+^ T cells is a major challenge in curing HIV infection^2^. Moreover, discontinuation of the antiretroviral therapy can lead to HIV-1 resistance to the antiretroviral therapy regimen with a concomitant rapid rebound of viral loads leading to disease progression toward AIDS^3^.

Recent reports of three patients show they have been cured of HIV-1 infection by the transplantation of hematopoietic stem cells from allogenic donors^4–6^. The stem cells have a homozygous mutation in the CCR5 gene (CCR5Δ32/Δ32), this results in no CCR5 on their cell membrane, and confers resistance to CCR5-tropic HIV strains. There are a few populations that have naturally occurring CCR5Δ32. The most abundant CCR5 gene mutation is the heterozygous mutation which presents at ~ 10%. However, the homozygous mutation frequency is only ~ 1% as presenting in the Caucasian population (European descent), while this allele is almost absent in Africans, Native Americans, and Asians^7^. Due to the limited distribution of the homozygous CCR5Δ32 genotype donors, alternative approaches to obtain sources of CCR5Δ32/Δ32 HSCs are required.

Several genome-editing tools have been employed to modify and eliminate the human CCR5 gene expression. Several different mechanisms have been targeted to affect the expression, including Zinc Finger Nucleases (ZFNs)^8^, Transcription Activator-Like Effector Nucleases (TALENs)^9^, and the Clustered Regularly Interspaced Short Palindromic Repeats (CRISPR)/CRISPR-associated (Cas) system^10,11^. ZFNs and TALENs rely on DNA-binding domains which are generated through libraries to target a specific DNA locus of interest, both are linked with the endonuclease FokI to induce DNA double strand breaks^12^. In contrast, CRISPR/Cas system does not encode the DNA-binding domain fused onto the Cas protein that generates the DNA double strand breaks. The genomic locus of interest is identified by a targeting CRISPR RNA (crRNA) and requires a trans-activating CRISPR RNA (tracrRNA) to facilitate binding of the Cas protein to mediate a DNA double strand break^13^. The main benefit of using the CRISPR/Cas system is simplicity of target design, efficiency of utilization, as well as flexibility of the technique. These gene targeting technologies have increasingly led to promising translational studies and clinical trials^14^.

The use of CRISPR/Cas9 system as a gene editing tool to mediate CCR5 knockout hematopoietic stem and progenitor cells (HSPCs) has successfully been employed in clinical trials (ClinicalTrials.gov identifier NCT03164135)^14^. A patient with HIV-1 infection and acute lymphoblastic leukemia was transplanted with HSPCs containing CRISPR/Cas9 ablated CCR5 gene. The single guide RNAs (sgRNAs) were screened to eliminate off-target potentials and were designed with high cleavage efficiency to pair at the beginning of the first exon of human CCR5 gene at the Δ32 mutation site. The CCR5-knockout HSPCs engrafted and displayed the donor cells CCR5 ablation which persisted in peripheral blood for more than 19 months. No gene-editing-related adverse events were detected. This finding holds the promise of finding alternative sources of CCR5Δ32/Δ32 HSC donors. However, the ablation of CCR5 might not be enough to protect against the X4 tropic HIV-1 which is a common HIV-1 strain infecting human CD4^+^ T cells and arises late in infection in about 50% of HIV patients^15^. Strategies for inhibiting HIV-1 in both R5 and X4 tropisms are still required.

In the present study, we developed a lentiviral vector construct to express C46, a membrane anchored HIV-1 fusion inhibitor, to combine with the CRISPR/Cas9 mediated knockout CCR5 to obtain a better outcome of protection in broad HIV-1 infections.

## Results

The MT4CCR5 cell line was used as the model to investigate the combination of two anti-HIV-1 entry effects. The CCR5 expression was stably ablated by the action of the CRISPR/Cas9 non-viral system mediated CCR5 knockout. The percentage of cells expressing the HIV-1 C46 fusion inhibitor by the lentiviral vector construct was increased by puromycin selection. These cells provided protection against cell death and reduced HIV-1 replication in both R5 and X4 HIV-1 strains after HIV-1 challenge.

### Generation of cellular knockout CCR5 by CRISPR/Cas9

To disrupt human CCR5 gene expression in MT4CCR5 cells, we adapted the method of knockout CCR5 by ribonucleoprotein (RNP) complex mediated CRISPR/Cas9 genome editing system from ClinicalTrials.gov Identifier: NCT03164135. The MT4CCR5 cells were nucleofected with two doses of in-house Cas 9 proteins and a pair of single guide RNAs (sgRNAs) to specifically target the first exon of the CCR5 gene. These sgRNAs have been proven to provide high gene-editing efficacy, low off-target effects, as well as long-term disruption of CCR5 to achieve significant resistant to the R5-tropic HIV-1 strain in vivo, in animal models, and in patients with HIV-1 and acute lymphocytic leukemia^11,14^.

**Figure 1:**
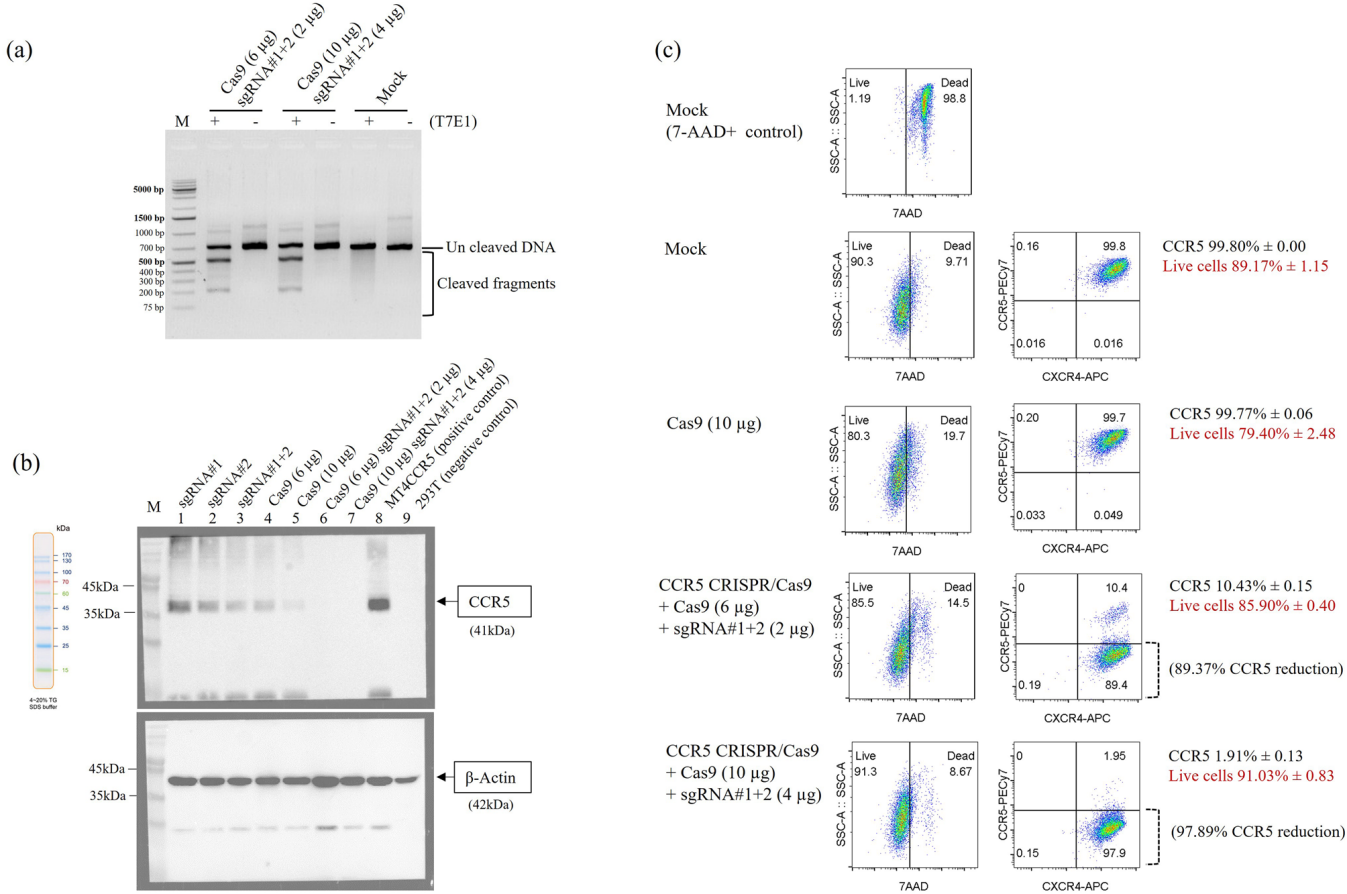
Efficient CCR5 ablation in human CCR5 T-cell line mediated by CCR5 CRISPR/Cas9. MT4CCR5 cells were evaluated for CCR5 cleavage efficiency and protein expression at 3 days post-nucleofection (**a**) The cleavage efficiency was determined using T7EI assay. A representative assay demonstrates efficient disruption of human CCR5 gene disruption by the CCR5 CRISPR/Cas9 in MT4CCR5 cell line. The PCR products (~ 647 bp) were digested into two fragments (~ 465 and ~ 182 bp), indicating effective CCR5 disruption. Mock is non-transfection control. (**b**) Reduction of CCR5 expression determined by SDS-PAGE and western blot analysis. Whole cell lysates were analysed for CCR5 protein by anti-human CCR5 antibodies. MT4CCR5 cell lines transfected with only sgRNA#1, #2, and combined #1 and #2 are shown in Lane 1–3. The cells transfected with only in-house Cas9 at 6 µg is shown in Lane 4, and 10 µg are shown in Lane 5. The cells transfected with in-house Cas9 protein (6 µg) and sgRNA#1 and #2 (each 2 µg) are shown in Lane 6. The cells transfected with in-house Cas9 protein (10 µg) and sgRNA#1 and #2 (each 4 µg) are shown in Lane 7. The mock control shown in Lane 8 represents no CCR5 disruption. 293T cell lysate utilizes for anti-CCR5 negative control shown in Lane 9. β-actin was included as the loading control. Following the determination of CCR5 protein expression, the same membrane was stripped and subsequently stained with the anti-β-Actin antibody. (**c**) Reduction of CCR5 expression measured by flow cytometry. Representative flow cytometry plots illustrate live cell population gating (7AAD^−^ cells) in the left panel, followed by analysis of CCR5 expression by staining with an anti-CCR5 antibody conjugated with PECy7 in the right panel. Additionally, negative control for CCR5 reduction involved staining CXCR4, the other HIV-1 co-receptor, with anti-CXCR4 antibody conjugated with APC (CXCR4-APC). The numbers indicated on the right side represent percentages of live cells and CCR5 gene expression levels, with mean ± SD calculated from triplicate experiments. Positive control for death cells were conducted by exposing MT4CCR5 cells to 56 °C for 30 min before staining, followed by determination of the 7AAD^+^ population.

We would like to observe dose dependent effects of CRISPR/Cas9 RNP complex to knockout CCR5 gene. The first dose to form an RNP complex was 6 µg of in-house Cas9 protein plus 2 µg of each sgRNA. The second dose was 10 µg of in-house Cas9 protein plus 4 µg of each sgRNA. The amounts of Cas9 and sgRNAs were applied according to the publication of Xu et al.^11,14^. We used 6 µg of Cas9 with 2 µg of sgRNA1# and 2 µg of sgRNA2# (total 4 µg of sgRNAs) following the ratio used with human CD34 + cells reported by Xu et al.^14^. We have defined this combination of Cas9 and sgRNAs as the first dose. Additionally, we used 10 µg of Cas9, as described in the publication by Xu et al.^11^, which investigated its use in K562 cell lines. We adapted this protocol by increasing the amount of sgRNAs to 4 µg of sgRNA1# and 4 µg of sgRNA2# (total 8 µg of sgRNAs). We have defined this combination of Cas9 and sgRNAs as the second dose. The cells were assessed for CCR5 cleavage efficiency and protein expression at 3 days post-nucleofection. We found no difference in cleavage efficiency of the pair of sgRNA truncated with the in-house Cas9 protein in both doses determined by T7 endonucleases I (T7E1) assay (Fig. 1a, and Supplementary Information 1).

The investigation of CCR5 protein expression levels by SDS-PAGE and western blotting (WB) revealed that the RNP complex, consisting of both sgRNAs and in-house Cas9 protein, significantly reduced CCR5 protein expression. While cells treated only with sgRNA1# (4 µg), sgRNA2# (4 µg), a combination of sgRNA1# (4 µg) and sgRNA2# (4 µg), in-house Cas9 protein (6 µg), or in-house Cas9 protein (10 µg) showed slight reductions in CCR5 protein expression as showed in Fig. 1b. This reduction detected in SDS-PAGE and WB analysis can be attributed to decreased cell viability post-nucleofection, as determined by 7AAD staining analyzed by flow cytometry analysis (Supplementary Information 3). The flow cytometry analysis of CCR5 expression in only live cell populations revealed no significant differences in CCR5 expression levels, approximately 99%, compared to mock cells, across all groups treated with individual sgRNAs or in-house Cas9 protein (Supplementary Information 3).

We found that the two doses of CRISPR/Cas9 RNP complex showed an approximately range between 77.50 and 98.40% cell viability after nucleofection, as determined in 7AAD^-^ cell population analysis by flow cytometry (Fig. 1c and Supplementary information 3). The dose dependence of the CRISPR/Cas9 RNP complex was shown in MT4CCR5 cells nucleofected with the first dose of RNP complex, which composed of 6 µg of in-house Cas9 protein and 2 µg of each sgRNA displayed a CCR5 expression level of 10.43% ± 0.15 (mean ± SD), representing an 89.37% reduction compared to the mock control (99.80% ± 0.00, mean ± SD). Conversely, cells receiving the second dose of RNP complex, comprised of 10 µg of in-house Cas9 protein and 4 µg of each sgRNA exhibited a CCR5 expression level of 1.91% ± 0.13 (mean ± SD), corresponding to a 97.89% reduction in CCR5 expression (Fig. 1c and Supplementary Information 3).

We chose the MT4CCR5 cells which showed the greatest CCR5 reduction to combine with the C46 HIV-1 fusion inhibitor. This would allow broad-spectrum inhibition of HIV-1 infection that included not only R5 tropism but also X4 tropism.

### Construction of lentiviral vector for the expression of C46 HIV-1 fusion inhibitor

The gene encoding the C46 HIV-1 fusion inhibitor was cloned into a third-generation lentiviral vector for stable expression of the inhibitor on the cell surface of MT4CCR5 cells. This antiviral fusion inhibitor is a cell membrane-anchored C-peptide that competes with the viral C-terminal heptad repeats binding to the N-terminal heptad repeat domain of the HIV-1 gp41 intermediate structure, subsequently blocking the HIV-1 membrane fusion and entry^16^. The C46 HIV-1 fusion inhibitor has been shown to inhibit both R5- and X4-tropic HIV-1 strains in various systems including tissue cultures and animal models^17–21^.

**Figure 2:**
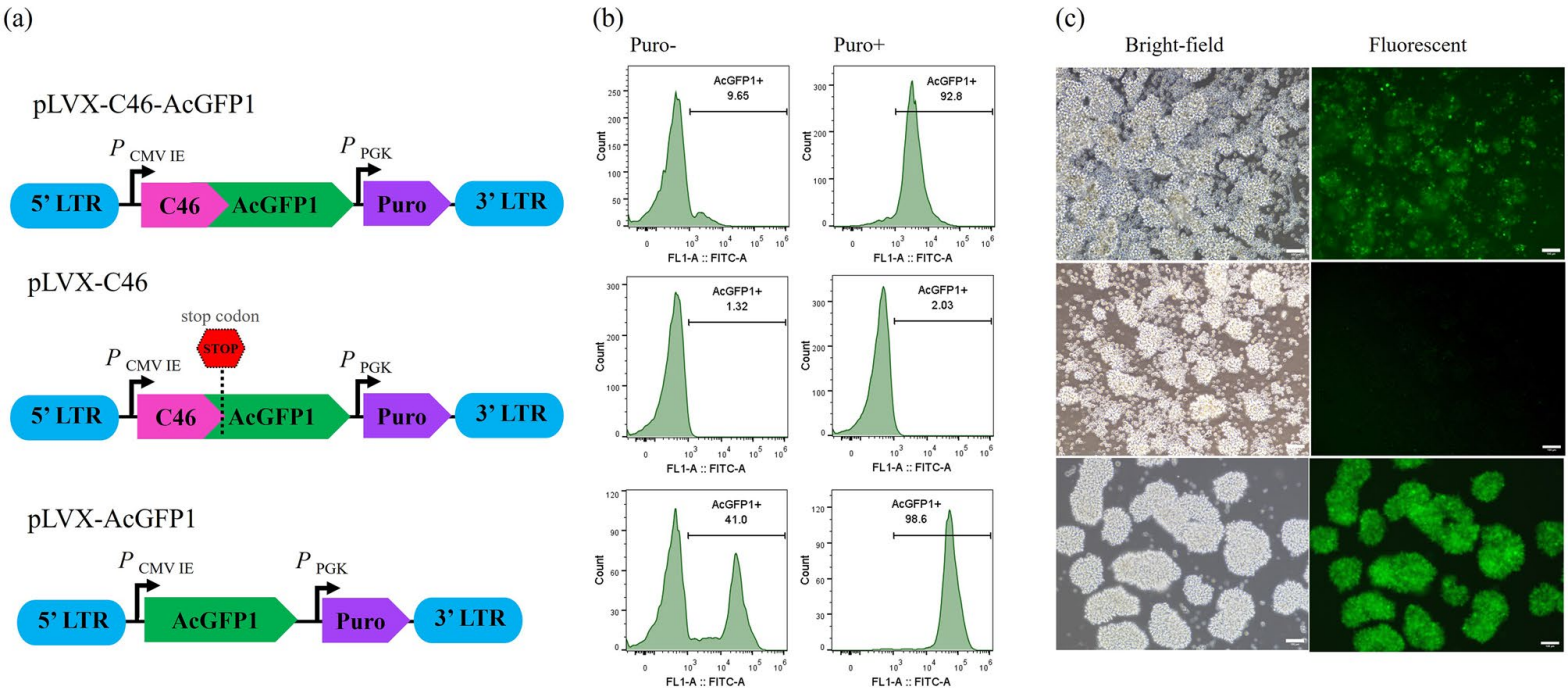
Construction of lentiviral vector for expressing C46 HIV-1 fusion inhibitor. (**a**) Schematics of the lentiviral vector constructions for expressing C46 HIV-1 fusion inhibitor while expressing green fluorescent protein (pLVX-C46-AcGFP1), top panel; the construct not expressing green fluorescent protein by adding stop codon (pLVX-C46), middle panel; and non-anti-viral control (pLVX-AcGFP1), lower panel. LTR: Long terminal repeat, *P*_CMV IE_: CMV IE promoter, *P*_PGK_: PGK promoter, Puro: puromycin resistant gene. (**b**) Representative flow cytometry histograms show AcGFP1 + expression after the lentiviral vector transduction in MT4CCR5. The cells transduced with the lentiviral vector at MOI of 0.1. The AcGFP1 + expression at day 3 post-transduction before puromycin selection is shown in the left panel, while the right panel illustrates the expression after 7 days post-puromycin selection. (**c**) Representative cell morphology and AcGFP1 + expression after the lentiviral vector transduction and puromycin selection were investigated by fluorescence microscopy.

We constructed two versions of the anti-HIV-1 lentiviral vectors expressing the C46 peptide. pLVX-C46-AcGFP1 was the lentiviral vector that expressed the C46 HIV-1 fusion inhibitor fused to AcGFP1 (*Aequorea coerulescens* GFP) under the control of a CMV EI promoter (*P*_CMV EI_). AcGFP1 is a monomeric green fluorescent protein utilized for tracking cells that express the C46 HIV-1 fusion inhibitor after lentiviral transduction. The other version was pLVX-C46. In this version we added a stop codon between the C46 gene and AcAGFP1 to express only the C46 HIV-1 fusion inhibitor for future therapeutic purposes. The pLVX-AcGFP1 was utilized as the non-antiviral effect control. Each lentiviral construct contained puromycin resistant gene encoded by PKG promotor (*P*_PKG_) for the advantage of antibiotic post-transduction selection (Fig. 2a). The transduced cells were evaluated for transduction efficiency at 3 days post-transduction by flow cytometry and fluorescent microscope to determine AcGFP1 expression. The puromycin selection of the transduced cells yielded only cells expressing the transgenes. We found that at 7 days of maintenance in puromycin 1 µg/ml, the non-transduced negative control cells (mock MT4CCR5 cells) died completely. Whereas the percentage of AcGFP1 + cells dramatically increased in the cells transduced with pLVX-C46-AcGFP1 from 9.65 to 92.8%, and pLVX-AcGFP1 from 41.0 to 98.60% (Fig. 2b). The transduced cells with pLVX-C46 were observed to contain a few green fluorescent positive cells. All lentiviral transduced cells survived post puromycin selection determined by trypan blue staining and by observing the healthy cell morphology under a bright field fluorescent microscope (Fig. 2c). TaqMan Real-Time PCR was employed to confirm the presence of C46 HIV-1 fusion inhibitor gene in pLVX-C46 and pLVX-C46-AcGFP1 transduced cells. Both pLVX-C46 and pLVX-C46-AcGFP1 transduced cells gave a C46 gene amplification signal at Ct 29.86 ± 0.14 and 29.31 ± 1.14 (mean ± SD) respectively. Whereas no signal was detected for the mock non-transduced cell control and the pLVX-AcGFP1 non-antiviral control. The beta globin housekeeping gene was used as an internal DNA control. Every sample yielded a similar amount of beta globin gene within the range of Ct 24.16 ± 0.74 to 24.88 ± 0.46 (mean ± SD) (Table 1).

**Table 1:** C46 gene integration levels in transduced cells.

	C46 (Ct)	Beta globin (Ct)
MT4CCR5/pLVX-C46-AcGFP1	29.31 ± 1.14	24.16 ± 0.74
MT4CCR5/pLVX-C46	29.86 ± 0.14	24.77 ± 0.09
MT4CCR5/pLVX-AcGFP1 (-control)	N/A	24.88 ± 0.14
MT4CCR5 (-control)	N/A	24.87 ± 0.03
C46 plasmid + MT4CCR5 (+ control)	8.60 ± 0.68	24.88 ± 0.46

The C46 gene integration in MT4CCR5 genome after lentiviral transduction and puromycin selection were evaluated by real-time PCR from the extracted genomic DNA using primers and probes specific to C46 gene. The cellular beta globin gene was used as the loading internal control. The levels of genes are represented in Ct values, Mean ± SD, N/A = non-detected.

For future applications in hematopoietic stem cell transplantation, even though the CMV IE promoter is a widely used due to its strong and constitutive activity in driving the expression of transgenes in mammalian cell lines^22^, its activity is moderately inefficient in primary human T cells and cord blood CD34 + cells in vitro^23^. Nonetheless, CMV IE promoters continue to function after differentiation of the transduced CD34 + cells into myelomonocytic and dendritic cell lineages, suggesting that the differentiation process did not silence the promoter activity^23^. Another concern regarding the use of CMV promoters is that they are prone to attenuation of expression and silencing within a few weeks after gene transfer^24^. Therefore, careful consideration of promoter options and codon usages for optimizing transgene expressions is necessary to ensure sustained gene expression over the long-term^25,26^.

### The combined CCR5 CRISPR/Cas9 and C46 HIV-1 fusion inhibitor

**Figure 3:**
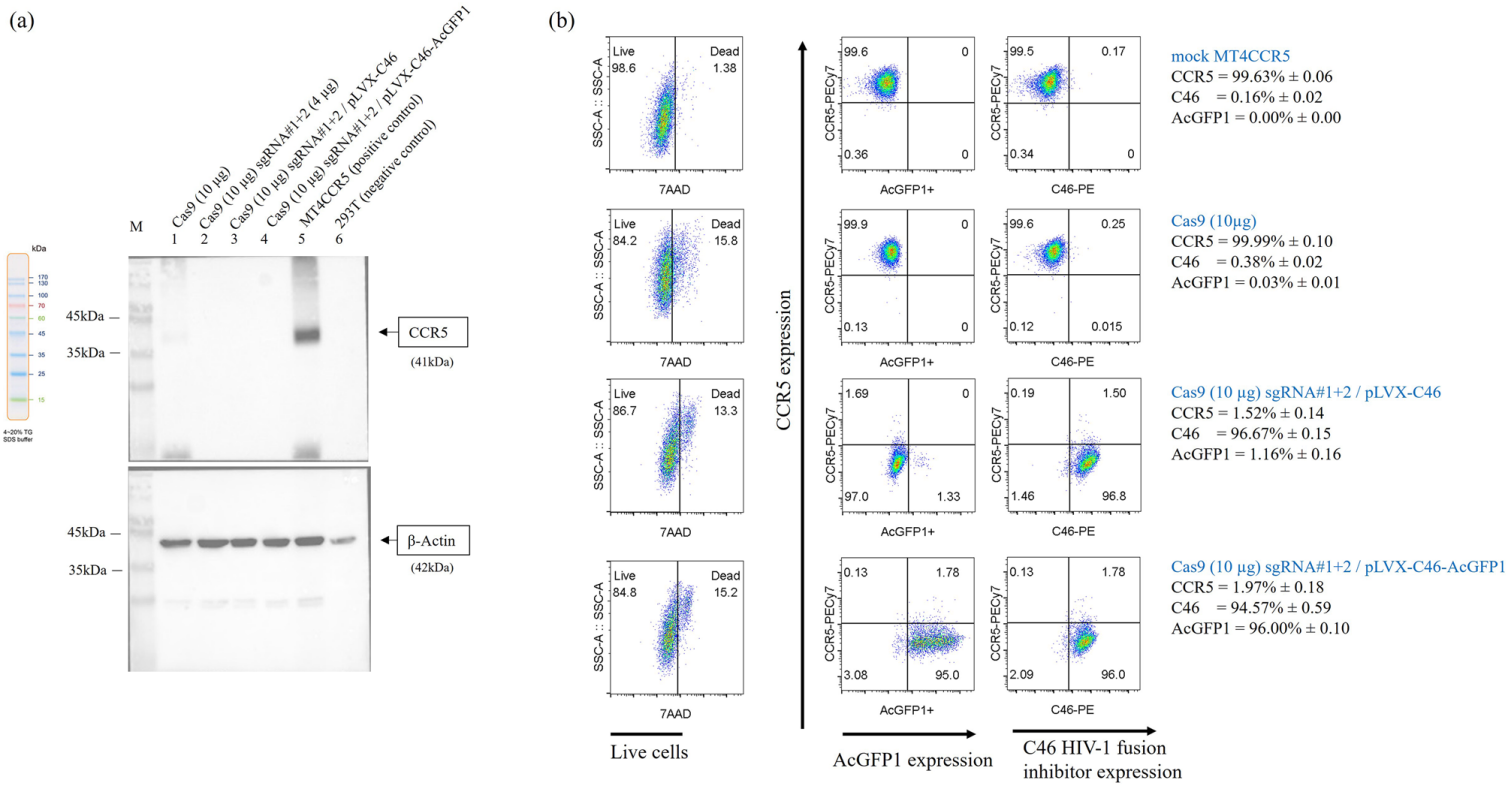
The combined CCR5 CRISPR/Cas9 and C46 HIV-1 fusion inhibitor. (**a**) Western blot analysis of CCR5 expression following combined anti-HIV-1 gene therapy. The whole cell lysate was probed and analyzed for CCR5 protein using anti-human CCR5 antibodies. MT4CCR5 cell lines transfected with only in-house Cas9 protein (10 µg) are shown in Lane 1. Cells transfected with CRISPR/Cas9 to knockout CCR5 with in-house Cas9 protein (10 µg) and sgRNA#1 and #2 (each 4 µg) are displayed in Lane 2. The combined CRISPR/Cas9 knockout of CCR5 with pLVX-C46 without AcGFP1 is shown in Lane 3, while Lane 4 represents the combined CRISPR/Cas9 knockout of CCR5 with pLVX-C46-AcGFP1. Mock is utilized as the positive control for anti-CCR5 staining (Lane 5), and 293T cell lysate serves as the negative control for anti-CCR5 staining (Lane 6). (**b**) Flow cytometry analysis of CCR5 reduction and the expression of the C46 HIV-1 fusion inhibitor on the cell membrane. The combined CRISPR/Cas9 for CCR5 and C46 HIV-1 fusion inhibitor in cells containing the construct with AcGFP1 or without AcGFP1, along with those transfected with single in-house Cas9 protein (10 µg), and mock control cells, were stained for the analysis of CCR5 expression using anti-CCR5 antibodies conjugated with PECy7 (CCR5-PECy7). The expression of the C46 HIV-1 fusion inhibitor was determined by either the presence of AcGFP1 + or using the anti-2F5 antibody specific to the C46 peptide, followed by staining with anti-human IgG conjugated with PE (C46-PE). The flow cytometry plots depict representative data, with the numbers indicating the gene expression levels in percentage mean ± SD.

To provide a broad-spectrum inhibitory mechanism for anti-HIV-1 in CRISPR/Cas9 knockout CCR5 cells, the lentiviral vector encoding C46 HIV-1 fusion inhibitor; pLVX-C46 and pLVX-C46-AcGFP1, and the non-antiviral control, pLVX-AcGFP1, were transduced in the CCR5-knockout MT4CCR5 cells at MOI of 0.1. After 3 day-post transductions, cells were cultured in the medium containing of 1 µg/ml puromycin for 7 days. The combination of antiviral genes after selection were determined by SDS-PAGE and western blot and flow cytometry. The CCR5-knockout MT4CCR5 cells that received pLVX-C46 or pLVX-C46-AcGFP1 lentiviral vector still maintained CCR5 reduction with similar levels shown before transduction as determined by SDS-PAGE and western blotting (Fig. 3a and Supplementary Information 4).

To investigate the levels of CCR5 reduction and C46 expression levels simultaneously, the cells were stained with specific antibodies against CCR5 and C46 HIV-1 fusion inhibitor, followed by evaluation using flow cytometry. The anti-human CCR5 mAb conjugated with PECy7 was used for measuring CCR5 expression on the cell surface. The anti-HIV-1 gp41 mAb (2F5) followed by anti*-*Human IgG Fc conjugated with PE were used to detect the levels of C46 HIV-1 fusion inhibitor on the cell surface. The analysis revealed a reduction in CCR5 expression from 99.63% ± 0.06 (mean ± SD) observed in the mock MT4CCR5 cells to 1.52% ± 0.14 and 1.97% ± 0.18 (mean ± SD) in the CCR5 CRISPR/Cas9 cells transduced with pLVX-C46 and pLVX-C46-AcGFP1 lentiviral vectors, respectively. The expression levels of C46 HIV-1 fusion inhibitor after staining were 96.67% ± 0.15 and 94.57% ± 0.59 (mean ± SD) in the cells transduced with pLVX-C46 and pLVX-C46-AcGFP1 lentiviral vector, respectively. Moreover, the expression of C46 fused with AcGFP1 in the cells transduced with pLVX-C46-AcGFP1 lentiviral vector illustrated AcGFP1 expression 96.00% ± 0.10 (mean ± SD) in the panel of AcGFP1 + to confirmed that the stanning C46 on the cell surface is equal amount to C46-AcGFP1 + expression. In addition, the cells transduced with pLVX-C46 which added stop codon before AcGFP1 gene were stop the expression of AcGFP1 but not C46 expression, as determined by presenting only AcGFP1 + to 1.16% ± 0.16 (mean ± SD) (Fig. 3b and Supplementary Information 5).

Moreover, in cells transduced with the pLVX-C46-AcGFP1 lentiviral vector, the expression of C46 fused with AcGFP1 was confirmed by the expression of AcGFP1, which accounted for 96.00% ± 0.10 (mean ± SD) in the AcGFP1 + panel, indicating that the staining of C46 on the cell surface corresponds to an equal amount of C46-AcGFP1 + expression. Furthermore, cells transduced with the pLVX-C46, which included a stop codon before the AcGFP1 gene, halted the expression of AcGFP1 but not C46 expression. This was demonstrated by the presentation of only AcGFP1 + to 1.16% ± 0.16 (mean ± SD) and C46-PE to 96.67% ± 0.15 (mean ± SD) (Fig. 3b and Supplementary Information 5). Additionally, levels of CCR5, CXCR4, and CD4 expression, indicative of HIV-1 receptor and co-receptor levels, were assessed in cells treated with CRISPR/Cas9 knockout CCR5, pLVX-C46, or pLVX-C46-AcGFP1 using flow cytometry. All cells exhibited CD4 and CXCR4 expression levels exceeding 97.30%. However, cells treated with CRISPR/Cas9 knockout CCR5 showed a CCR5 expression level of 1.94%, while the others exhibited levels exceeding 98.8% (Supplementary Information 6).

### Anti-viral effects of the combined anti-HIV-1 therapeutic genes

**Figure 4:**
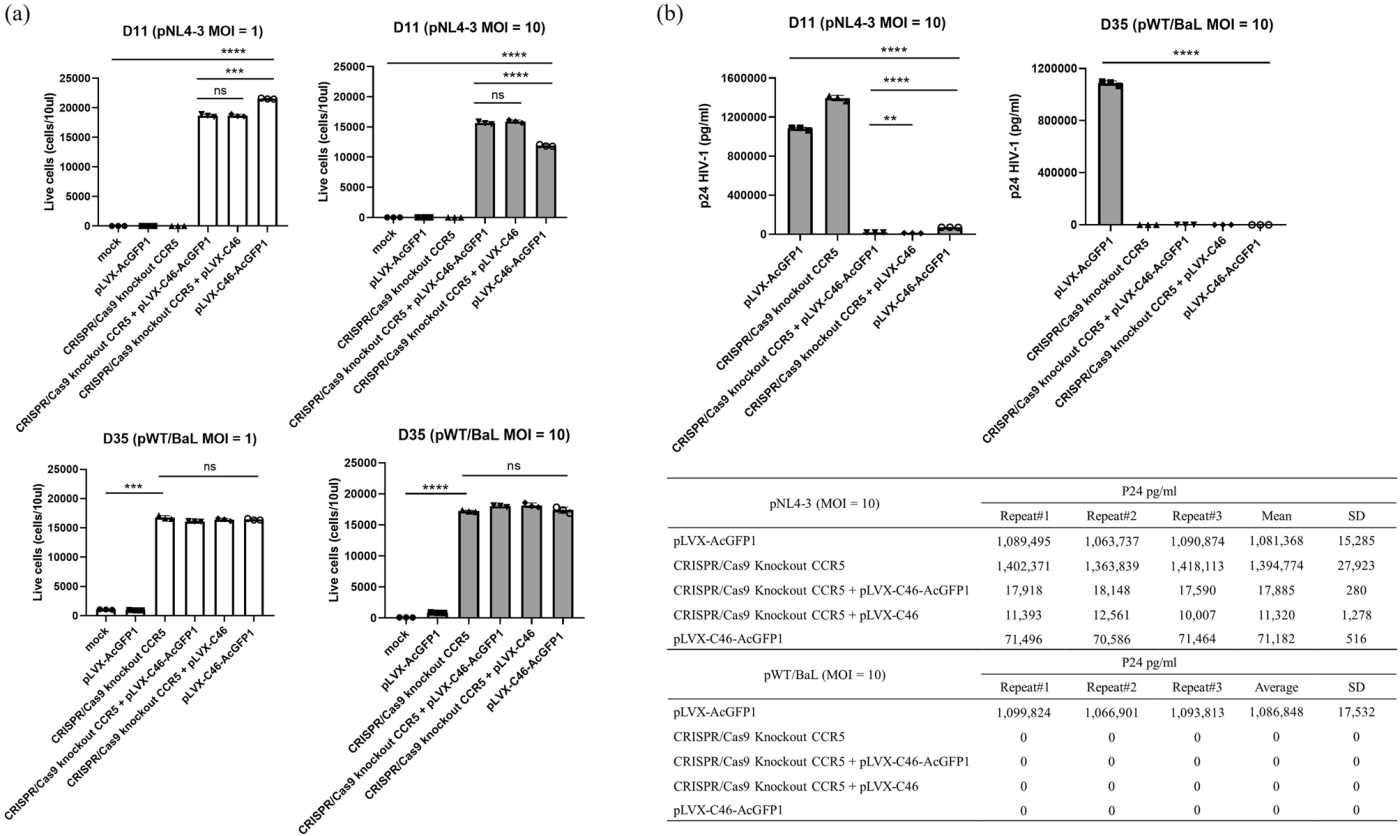
Anti-viral effects of the combined CCR5 CRISPR/Cas9 and C46 HIV-1 fusion inhibitor. HIV-1 inhibition in vitro. The non-antiviral control cells, mock and pLVX-AcGFP1 transduced MT4CCR5 cell line, the single anti-HIV-1 gene either CCR5 CRISPR/Cas9 or pLVX-C46-AcGFP1, and the combined CCR5 CRISPR/Cas9 and C46 HIV-1 fusion inhibitor were challenged with R5-tropic HIV-1_BaL_ (MOI 1 and 10) or X4-tropic HIV-1_NL4-3_ (MOI 1 and 10), respectively. Cell viability and viral replication in culture supernatant were evaluated at day 11 and day 35 post HIV-1 challenge. (**a**) Number of live cells were measured by 7AAD^-^ cells and analyzed by flow cytometry as shown in the figure and Supplementary Information 7. (**b**) Levels of HIV-1 p24 production (pg/ml) were measured in culture supernatant using p24 HIV-1 ELISA. The left panel showed p24 levels at day 11 post HIV-1_NL4-3_ infection (MOI of 10). The right panel showed p24 levels at day 35 post HIV-1_BaL_ infecteion (MOI of 10). Unpaired t-test with Welch’s correction was performed to calculate significance. Bars and error bars show Mean ± SD. **p < 0.01, ***p < 0.001, and ****p < 0.0001, ns = not significant. The table in the lower right panel displays the levels of HIV-1 p24 production in the culture supernatant after HIV-1 infection, measured by p24 HIV-1 ELISA (pg/ml). The upper table presents p24 levels at day 11 post HIV-1_NL4-3_ infection (MOI of 10), while the lower table shows p24 levels at day 35 post HIV-1_BaL_ infection (MOI of 10).

To examine resistant to both R5- and X4-tropic HIV-1 infections, the CRISPR*/*Cas9 knockout CCR5 in MT4CCR5 cells, the pLVX-C46-AcGFP1 transduced cells, and combined C46 HIV-1 fusion inhibitor with the CCR5 knockout cells were challenged with CCR5 (R5)-tropic HIV-1_BaL_ or with CXCR4 (X4)-tropic HIV-1_NL4-3_. The viral challenges at MOI of 1 represented the low levels of HIV-1 infection and at MOI of 10 represented the high levels of HIV-1 infection. The pLVX-AcGFP1 vector-transduced MT4CCR5 and mock MT4CCR5 were used as non-antiviral negative control. The cell death post-viral infections were verified with 7AAD^+^ cell populations and live cells with 7AAD^-^ cell population analysis by flow cytometry (Supplementary Information 7). We found that, the CRISPR/Cas9 knockout CCR5 cells protected against cell death only the R5-tropic HIV-1_BaL_ infections at both low and high levels of viral infection but did not protect from the X4-tropic HIV-1_NL4-3_. The CRISPR*/*Cas9 knockout CCR5 in MT4CCR5 cells all died by day 11 post X4-tropic HIV-1_NL4-3_ infection. Both MOI infections showed a similar response and died in the same time frame as the negative control cells. The CRISPR/Cas9 knockout CCR5 combined with C46 HIV-1 fusion inhibitor with/without AcGFP1 protected against cell death from both R5-tropic HIV-1_BaL_ and X4-tropic HIV-1_NL4-3_ infections. It was noted that the single or combined anti-viral therapeutic genes prevented cell death at both MOI of R5-tropic HIV-1_BaL_ infection. (Fig. 4a).

The levels of HIV-1 replication in the culture supernatant after HIV-1 infections at MOI of 10 were measured by HIV-1 p24 ELISA. We detected no viral replication for the cells that received single or combined anti-HIV genes up to 35 days post the R5-tropic HIV-1_BaL_ infection_._ This result suggests that either the individual effect of CRISPR*/*Cas9 knockout CCR5 or the single expression of C46 fusion HIV-1 inhibitor would likely be sufficient to protect against the R5-tropic HIV-1_Bal_ infection. Thus, the combined treatments continue to combat the R5-tropic HIV-1_BaL_ infection. Cells challenged with the X4-tropic HIV-1_NL4-3_ showed high levels of p24 HIV-1 at day 11 post-infection in the single CRISPR*/*Cas9 knockout CCR5 (1,394,774 pg/ml ± 27,923; mean ± SD) and the non-anti-viral control cells (1,081,368 pg/ml ± 15,285; mean ± SD), suggesting that only the effects of CRISPR*/*Cas9 knockout CCR5 alone do not prevent the X4-tropic HIV-1_NL4-3_ infection. However, the cells transduced with the single pLVX-C46-AcGFP1 showed p24 HIV-1 levels of 71,182 pg/ml ± 516 (mean ± SD). This shows a 15.2-fold reduction in the X4-tropic viral replication compared to the non-anti-viral control. The combined CRISPR/Cas9 knockout CCR5 with C46 HIV-1 fusion inhibitor with/without AcGFP1 cells showed p24 HIV-1 levels of 17,885 pg/ml ± 280 (mean ± SD) and 11,320 pg/ml ± 1278 (mean ± SD), respectively. This is a reduced viral replication of 3.9- and 6.3-fold compared to the single pLVX-C46-AcGFP1 transduced cells and a reduction of 60.5- and 95.5-fold compared to the non-anti-viral control, respectively (Fig. 4b). The expression of C46 fusion HIV-1 inhibitor alone in cells would likely be sufficient to against both R5- and X4-tropic strains of HIV-1, as this fusion inhibitor can effectively block HIV-1 entry in both strains^18,20^. The potential efficacy of using the lentiviral system with C46 alone to protect HIV-1 infection may reduce potential off-target effects associated with the CRISPR/Cas9 system. However, in an in vivo setting, we may not be able to control the levels of HIV-1 replication. Overwhelming HIV-1 replication could result in insufficient expression of C46 on the surface of target cells to prevent breakthrough HIV-1 infections. Nonetheless, the combined approach of using the C46 with the CRISPR/Cas9 knockout CCR5 still offers advantages in at least removing the CCR5 co-receptor as an entry site for R5-tropic HIV-1 infection. It is worth noting that the combined anti-viral therapeutic genes provided greater protection against the HIV-1 strain replications.

## Discussion

CRISPR/Cas9 as an effective genome-editing tool brings new hope for the eradication of HIV-1 infection. This report illustrates a strategy for knocking out CCR5 gene, presenting as an alternative approach to mimic the naturally homozygous CCR5 Δ32 mutation^11,27,28^. The ablation of CCR5 HIV-1 co-receptor can prevent only CCR5 tropic HIV-1 strains. Other crucial co-receptors have also been identified such as CXCR4, which is an entry pathway for CXCR4-tropic HIV-1 strains^21^. Gene-editing of CXCR4 has also been a target for a curative solution for the CXCR4-tropic HIV-1 infections. Ablation of CXCR4 by CRISPR/Cas9 system was shown to be feasible in cell lines and primary CD4^+^ T cells and conferred resistance to HIV-1 infection in vitro^29^. In a humanized mouse model, CRISPR/Cas9-mediated reduction significantly decreased the surface expression of the CCR5 and CXCR4 co-receptors in primary CD4 + T cells, thereby establishing protection of the cells from HIV-1 infection by R5-tropic, X4-tropic, and dual R5/X4-tropic strains. However, disruptions of both CCR5 and CXCR4 were observed to give poor engraftment in Hu-PBMC mouse bone marrow^30^. Of note, CXCR4 plays an important role in retainment of HSPCs in stem cell niches inside bone marrow and controls the migration of these cells to peripheral blood^31^. Therefore, disrupting CXCR4 may abate engraftment of the modified cells and disturb the maintenance of transplanted cells in bone marrow^32^.

In this study, we aim to combat both R5- and X4-tropic strains for broad-spectrum protection. We adapted a system to knockout CCR5 expression from a clinical trial study (ClinicalTrials.gov identifier NCT03164135)^14^. This system in combination with the anti-HIV-1 effects from C46 HIV-1 fusion inhibitor provides better protection against both R5- and X4- HIV-1 tropic strains^16^.

To generate the knockout CCR5, a pair of sgRNA were selected to target human CCR5 gene to produce efficient cleavage activity and eliminate off-target effects^11,14^. The cleavage of the CRISPR/Cas9 system was delivered by an RNP complex composed of an in-house Cas9 protein and a pair of sgRNA#1 and #2. The amount of in-house Cas9 protein was 6 µg with each sgRNA 2 µg which provided a level of CCR5 suppression that was reduced to ~ 11% on the cell membranes. Whereas an amount of in-house Cas9 protein of 10 µg and each sgRNA of 4 µg gave a CCR5 suppression similar to homozygote CCR5Δ32 which is the CCR5 expression levels are as low as ~ 1%. Determining CCR5 reduction using flow cytometry provides more detailed information compared to relying solely on the T7E1 assay^11^.

The blocking of X4-tropic HIV-1 infections is one of the functions of C46 HIV-1 fusion inhibitor. C46 is a membrane-anchored C-peptide specific to HIV-1 gp41^21^. C46 can trap HIV-1 virions at the cell surface and prevent the transfer of trapped virions to non-transduced neighboring cells^33^. C46 has been characterized in in vitro and in vivo settings including animal models and clinical trials to demonstrate significant protection from a broad range of HIV-1 strains including R5- and X4-HIV-1 tropic strains ^16,34–36^. We utilized the anti-HIV-1 effects of C46 HIV-1 fusion inhibitor through a construction of the third-generation lentiviral vector containing puromycin selection, instead of using a retroviral vector^16^ or a lentiviral vector without selection ability^33^. This drug selection provides the benefit of allowing only stably transgenic cells to survive to achieve significant levels of inhibition of HIV-1 infection^37,38^. The low efficiency of transduced cells before puromycin selection in our system may result from the use of a low MOI for transduction. Enhancing transduction efficiency can be achieved by using higher viral titers through lentiviral particle concentration via ultracentrifugation and using lentiviral transduction enhancers^39^. Additionally, transduction efficiency may be improved by removing protein debris from vector production processes, which can be toxic to target cells, through anion exchange HPLC purification^40^.

Administration of puromycin selection for gene-modified cells in clinical trials exhibit several challenges and limitations related to safety concerns. For future clinical applications, safer and more efficient strategies are needed for selecting anti-HIV gene-modified cells in vivo. The use of O^6^BG/BCNU selection of cells expressing mgmtP140K presents a promising alternative^41^. This strategy leverages the selective toxicity of O6-benzylguanine (O6BG) and BCUN in methylguanine methyltransferase (mgmtP140K) gene-modified cells, rendering them resistant to the cytotoxic effects of O^6^BG/BCNU and enhancing stable gene marking in mice^42^, canine^43^, and nonhuman primates^41,44^. Additionally, the use of 6-thioguanine (6TG) selection for cells knocking down hypoxanthine–guanine phosphoribosyl transferase (HPRT) expression offers another promising approach. The utilize 6TG significantly selects and enhances the engraftment of HPRT-knockdown anti-HIV-1 gene-modified cells in humanized bone marrow/liver/thymus (huBLT) mice^45^.

The combined anti-viral effects of both CCR5 CRISPR/Cas9 knockout and C46 HIV-1 fusion inhibitor provided a broad-spectrum effect for both R5- and X4-tropic infections in in vitro testing. The protections of R5-tropic infection are gained from the efficiency of the CCR5 CRISPR/Cas9 and the expression of C46 on cell membrane. The protection of X4-tropic infection is gained from the C46 fusion inhibitor. Utilization of the combination of these two anti-viral mechanisms in HIV-1 target cells should reduce the aggressive depletion of CD4^+^ T cells from X4-and R5-tropic HIV-1 infections^46^. The combined anti-viral effects of the C46 fusion inhibitor with a short hairpin RNA to CCR5 using a self-inactivating (SIN) lentiviral vector have been reported to stably downregulate CCR5, express C46, and protect against a broad range infection of HIV-1 strains in human cell lines, primary PBMCs, and human CD34^+^ HSPCs^18^. However, without transgene selection strategy may result in losing of gene modified cells in vivo. Additionally, over-expression of an shRNA is prone to off-target knockdown and mediated cytotoxicity^47^. Knocking out CCR5 using the RNP CRISPR/Cas9 system offers the advantage of rapidly exposing cells to Cas9 protein and sgRNA. This efficient method allows for quick modification of the target gene within a short period.

Our findings suggest that the combined expression of C46 HIV-1 fusion inhibitor on cell membranes of HIV-1 target cells in combination with CRISPR/Cas9-mediated CCR5 knockout strategy provide an alternative approach of genetic engineering for cellular resistance to a broad range of HIV-1 infections. This would be a promising option to use with stem cell-based gene therapy for an HIV-1 cure in future clinical trials. Autologous hematopoietic stem cell transplantation (HSCT), which involves using HIV-free hematopoietic stem/progenitor cells (HSPCs) containing the combined anti-HIV gene therapy, could serve as an alternative approach to curing HIV-1-infected individuals. Screening patients who have been on long-term highly active antiretroviral therapy (HAART) and have tested negative for proviral DNA in their HSPCs would be a good target group to investigate the functional cure proposed by this hypothesis in the future. Six HIV-1-infected individuals with prolonged viral suppression have been reported to be absent of intact proviral DNA in their peripheral CD34^+^ HSPCs^48^. These individuals could be candidates for further studies into the ability of gene-modification in autologous HSCT to lead to a definitive HIV cure.

However, a safety evaluation using adult HSPCs testing in in vitro and in vivo systems should be performed before clinical trials. Future research should focus on replicating the combined anti-HIV-1 gene therapy in normal and HIV-1 infected HSPCs to determine whether this approach impacts bone marrow engraftment, lineage development, long-term maintenance of the combined genes as well as does it raise any other safety concerns such as off-target effects and unintended chromosome loss^49^.

## Material and methods

### Cell culture

MT4CCR5 cells are a human CD4^+^ T cell-line modified to stably express CCR5 co-receptor after being transduced with a lentiviral vector expressing human CCR5 under the control of SFFV promoter. These cells are susceptible to both R5-and X4-tropic HIV-1. The MT4CCR5 cells were kindly provided by Dr. Koki Morizono (UCLA, Los Angeles). These cells were cultured in RPMI-1640 medium (Gibco) supplemented with 10% fetal bovine serum (Gibco), 2 mM L-glutamine, 100 units penicillin, and 100 μg/ml streptomycin (Gibco). The cell cultures were maintained in a humidified incubator at 37 °C and 5% CO_2_. HEK293T cell line (Clontech) was used for viral production and lentiviral titer. The cells were cultured in Dulbecco’s Modification of Eagles Medium (Hyclone) with the same supplements as MT4CCR5 cells.

### Lentiviral vector construction

The third-generation lentiviral vector, pLVX-AcGFP1-N1 (Clontech, Cat# 632154), was used as the backbone vector to transfer the C46 HIV-1 fusion inhibitor gene into the target cells. The C46 C-peptide fusion inhibitor sequence obtained from the publication of Felix G. Hermann et al., 2009^50^ were gene synthesized and cloned into pUC57 plasmid (Integrated DNA Technologies, Inc). The C46 HIV-1 fusion inhibitor sequence was designed to flank XhoI and EcoRI restriction sites to clone in the lentiviral vector, resulting pLVX-C46-AcGFP1. The pLVX-C46 plasmid was cloned by amplifying C46 HIV-1 fusion inhibitor gene without the expression of AcGFP1 from the synthesized pUC57 plasmid with the forward primer having the XhoI restriction site and the reverse primer adding a stop codon before the EcoRI restriction site. All construct lentiviral plasmids were confirmed to contain the C46 HIV-1 fusion inhibitor by restriction enzyme digestion and DNA sequencing.

### Lentiviral vector production

VSVG-pseudotyped lentiviral vector particles were produced in HEK293T cells, using four separate plasmids and the calcium phosphate transfection method, as previously described^51–53^. HEK293T cells were seeded on 10-cm cell culture dishes, and co-transfected with the construct lentiviral vector with the third-generation packaging plasmid system (Addgene plasmid #12251 (pMDLg/pRRE), #12253 (pRSV‐REV), #12259 (pMD2.G). Vector particles were harvested from the culture supernatant collected at 24 and 48 h. The viral vector titers, determined by infection of HEK293T cells with serial dilution of the samples, were expressed as the percentage of AcGFP1 + positive cells evaluated by flow cytometry.

### Lentiviral vector transduction and selection

MT4CCR5 cells and MT4CCR5 CRISPR/Cas9 knockout CCR5 cells (1 × 10^6^) were transduced with lentiviral vectors at a multiplicity of infection (MOI) of 0.1 with 8 μg/ml polybrene for 24 h. The transduced cells were seeded in a 6-well plate and incubated at 37 °C for 3 days before determining the percentage of transduced cells by flow cytometry. The transduced cells were enriched for those containing the lentiviral vectors by treating them with 1 µg/ml puromycin (Sigma‐Aldrich) in culture medium and refreshing the medium every 3–4 days.

### Cas9 protein expression and purification

3xNLS-SpCas9 plasmid (Addgene plasmid#114365), was transformed into ClearColi BL21(DE3) *E.coli* cells for protein expression. The recombinant protein was expressed and purified as previously published^54^. In detail, the cells were cultured in Luria–Bertani medium supplemented with 100 μg/ml of ampicillin at 37 °C until the cell density reached 0.6 at OD_600_ nm. Subsequently, protein expression was induced by adding 0.4 mM isopropyl-β-D-thiogalactopyranoside (IPTG), and the culture was maintained at 18 °C in a shaking incubator for 24 h. After induction, cells were centrifuged, and the cell pellets were stored at − 20 °C. To purify the Cas9 protein, the cell pellets were lysed in a buffer containing 20 mM Tris–HCl pH 7.5, 200 mM NaCl, 0.5 mg/ml lysozyme, and 1 mM DTT by incubating on ice for 10 min. The cells were then physically lysed by sonication and centrifuged at 15,000 rpm for 1 h at 4 °C. The supernatant was applied onto the first 1 ml of HisTrap FF prepacked column (Cytiva). After extensive washing with binding buffer (20 mM Tris–HCl pH 7.5, 200 mM NaCl) and binding buffer containing 20 mM Imidazole for 5 ml, the partially purified Cas9 protein was eluted with a buffer containing 200 mM imidazole. For the second purification step, hydrophobic interaction chromatography (HIC) column (phenyl sepharose HP) was utilized. The HIC column was pre-equilibrated with 20 mM Tris–HCl pH 7.5, 200 mM NaCl, and 2 M NaCl buffer. The elution sample from the first column was also supplemented with salt up to 2 M to promote protein binding. After applying the sample onto the column, Cas9 protein was eluted with a buffer containing 20 mM Tris–HCl pH 7.5, 200 mM NaCl, and 1 M NaCl. Finally, the Cas9 protein was further purified using gel filtration chromatography (superdex 200 increase 10/300 GL) as a third purification step. The purity of the final protein sample was evaluated by SDS-PAGE. The protein concentration was determined by the Bradford method using BSA as protein standard.

### Generation of CCR5 knockout by CRISPR/Cas9

The sgRNA1# sequences, 5′-ACTATGCTGCCGCCCAGT-3′; the sgRNA2# sequence 5′-CAGAAGGGGACAGTAAG-3′^14^. The sgRNAs targeting to CCR5 gene were synthesized with chemically modified nucleotides at the terminal positions at both the 5′ and 3′ ends were purchased from Integrated DNA Technologies, Inc. Ribonucleoprotein (RNP) complex was made by incubating 6 µg (36.81 pmol) or 10 µg (61.35 pmol) of the in-house purified Cas9 protein, 2 µg (61.86 pmol) or 4 µg (123.72 pmol) of sgRNA#1 or sgRNA#2 at room temperature for 20 min. Resuspended 1 × 10^6^ cells in 20 µl of SF cell line nucleofector solution, mixed with the RNP complex, and transferred into the Nucleocuvette (Lonza), program DC100. The nucleotransfection protocol was followed according to the manufacturer’s recommendations (Amaxa™ 4D-Nucleofector, Lonza).

### Flow cytometry

Cells were stained with monoclonal antibodies against human CCR5 (2D7: PECy7 labeled, eBioscience, Cat#557752), human CXCR4 (12G5: APC labeled, Biolegend, Cat#306510), human CD4 (OKT4: PE labeled, Biolegend, Cat#317410), according to the manufacturer’s instructions. C46 HIV-1 fusion inhibitor expression was measured by staining with 0.2 µg of anti-HIV-1 gp41 Monoclonal (2F5) (NIH AIDS Reagent Program, Cat#1475) followed by 50 µl of 1:500 of the secondary antibody (PE-anti-human Fc, Jackson Immunoresearch, cat#109-115-098). Isotype control antibodies were included with mouse IgG2b control-PE (ImmunoTools, Cat#21275534), mouse IgG1 control-APC (ImmunoTool, Cat#2185016), and mouse IgG1 control-PECy7 (Biolegand, Cat#400126). CCR5, CXCR4, CD4, C46 HIV-1 fusion inhibitor, and AcGFP1 expression were measured by flow cytometry using the CytoFLEX Flow Cytometer (Beckman Coulter). A live cell population defined by 7AAD^-^ staining (Biolegend, cat#420404) was subjected to single-cell population analysis for each antibody staining. Additionally, a positive control for dead cells was established by exposing MT4CCR5 cells to heat at 56 °C for 30 min before staining and determined by the 7AAD^+^ population. Data analysis for gene expression was performed with FlowJo version 10 software (BD Biosciences).

### C46 real-time PCR

The genomic DNA was isolated from cells using Invitrogen Purelink Genomic DNA Mini Kit (Invitrogen, cat#K182001) according to the manufacturer’s instructions. 25 ng of genomic DNA per reaction was validated with real-time PCR reactions set up in duplicate in 25 μl reaction volumes using Agilent Brilliant II master mix (Invitrogen) in a 96-well plate on CFX Connect Thermal Cycler platform (Bio-Rad). Taqman primer and probe sequences for this assay were as follows: C46 probe: 5′ 6-FAM/CA CTC CAC G/ZEN/C AGC ACT TCC GCT CG/IABkFQ′ 3, C46 forward primer: 5′ CAC AGC CTG ATC GAG GAG AG′ 3, C46 reverse primer: 5’ GTC CTG CCA CTG GTG GTG′ 3, β-globin probe: 5′ HEX/CT CCT GAG GAG AAG TCT GCC GTT ACT GCC /BHQ-2′ 3, β-Globin forward primer: 5′ CAA CCT CAA ACA GAC ACC ATG G′ 3, β-Globin reverse primer: 5′ TCC ACG TTC ACC TTG CCC’ 3. Thermocycling conditions were 50 °C 2 min, 95 °C 10 min, 40 × (95 °C 15 s; 60 °C 1 min)^18^.

### SDS-PAGE and Western blotting

Cells were washed with ice-cold PBS and lysed in radioimmunoprecipitation assay (RIPA) lysis buffer (Thermo-Fisher Scientific). The total protein concentration in the cell lysates was measured using the bicinchoninic acid assay (Thermo-Fisher Scientific). Subsequently, 30 µg of total protein was separated by SDS-PAGE and transferred onto a PVDF membrane (Thermo-Fisher Scientific). Blocking was carried out using 5% skim milk (Sigma) in 0.05% PBST for 24 h, followed by overnight incubation with the primary antibody (anti-CCR5 antibody; CUSABIO TECHNOLOGY, cat#CSB-PA006994). After washing the blots five times with 0.05% PBST, they were probed with the secondary antibody (Goat pAb to Rb IgG (HRP); AbCam, cat#ab205718) for 1 h and subsequently detected using ECL Western Blotting Substrate (Bio-Rad, cat#1705060) and scanned using the Odyssey InfraRed Imaging System (LI-COR BioSciences, Lincoln, NE). The blots were then stripped with mild stripping buffer (Thermo-Fisher Scientific), re-blocked, and re-probed with anti-β-Actin antibody (Abcam, cat#ab8227).

### T7 endonuclease I assay

Genomic DNA was extracted from cells using Invitrogen Purelink Genomic DNA Mini Kit (Invitrogen, cat#K182001) according to the manufacturer’s instructions. The target sequence was amplified using the Phusion high-fidelity DNA polymerase (Thermo Scientific, cat#F-530XL) and XhoI-CCR5 forward primers 5′-TGGACAGGGAAGCTAGCAGCAAA-3′ and EcoRI-CCR5 reverse primer 5′-TCACCACCCCAAAGG TGACCG-3′. The PCR products were annealed after purification. Next, 2 µl of hybridized DNA were subjected to digestion with 0.5 µL T7EI (New England Biolabs) in NE Buffer 2 for 30 min at 37 °C. Subsequently, the samples were loaded onto a 2% agarose gel electrophoresis with an equal amount of PCR product controls from non-edited samples.

### HIV-1 production

R5-tropic HIV-1_BaL_ virus and X4-tropic HIV-1_NL4-3_ virus were produced by transient transfection of pWT/BaL plasmid (NIH AIDS Reagent Program, cat#11414) or pNL4-3 plasmid (NIH AIDS Reagent Program, cat#114) into HEK293T cells. Monolayers of HEK293T cells (5 × 10^6^ cells per 10-cm dishes) were transfected with 10 µg of each plasmid using a calcium phosphate transfection method^51–53^. After 8 h, the transfection mixture was withdrawn, replaced by 10 ml of 10% FBS in DMEM medium. The transfected cells were incubated for 24 h. HIV-1 viruses were harvested from the culture supernatants and filtered through sterile syringe filters with a 0.45-µm pore size (Merck Millipore). HIV-1 samples were aliquoted and kept at − 80 °C. The virus titer was determined for the HIV viral load using the COBAS AMPLICOR HIV-1 Monitortest (version 1.5; Roche Molecular Systems, Branchburg, NJ).

### HIV-1 challenge

The 1 × 10^6^ cells were incubated with HIV-1 at MOI of 1 and 10 for 16 h. The cells were then washed three times with serum-free medium and resuspended in fresh growth medium. The infected cells were split into half at 3-day intervals, to maintain a cell density of approximately 10^6^ cells/ml. HIV-1 replication was monitored in culture supernatants, using HIV-1 p24 Simple Step ELISA kit (Abcam, cat#ab218268) and viral load assay, as described above. The cell pellets were kept determining cell viability by 7AAD staining (Biolegend, cat#420404) and flow cytometry.

### Statistical analysis

Data was obtained from triplicate experiments (n = 3). The data were analyzed, and standard deviations (SD) were calculated using the Prism 7 (GraphPad) statistical software program. Unpaired t-tests with Welch’s correction were used to calculate *p* values, and *p* < 0.05 was considered statistically significant.

### Supplementary Information

Supplementary Information.

## Data Availability

The data that support the findings of this study are available from the first author (W.K.) or the corresponding author (S.H.) upon reasonable request.
