# Supplementary material for: CRISPR/Cas9 genome editing of CCR5 combined with C46 HIV-1 fusion inhibitor for cellular resistant to R5 and X4 tropic HIV-1
Source: Sci Rep. 2024 May 13;14:10852. doi: 10.1038/s41598-024-61626-x (PMC11091187; doi:10.1038/s41598-024-61626-x)
Supplement: Supplementary file 1 — Supplementary Information. [file 41598_2024_61626_MOESM1_ESM.docx]

**
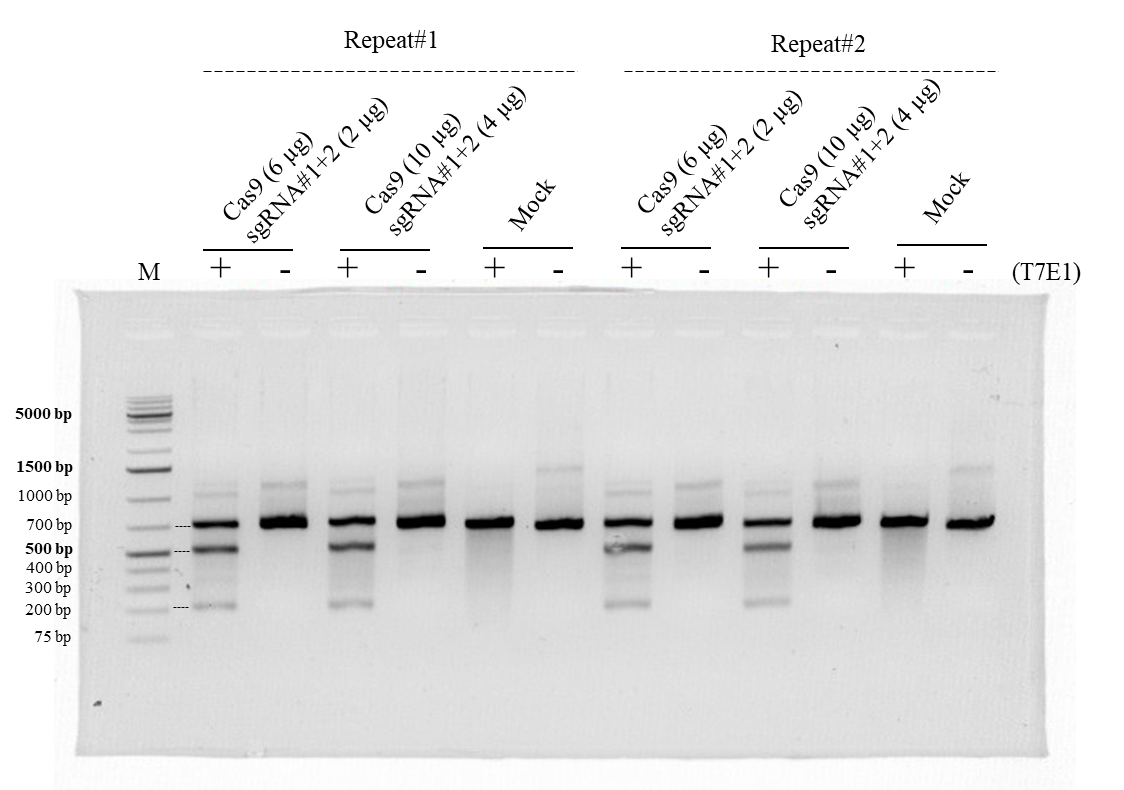
**

**Supplementary Information 1**

The full gel electrophoresis shows the cleavage efficiency after the CRISPR/Cas9 knockout CCR5 in MT4CCR5 cell lines. A representative assay illustrated the successful disruption of the human CCR5 gene, as determined by the T7 endonuclease I (T7EI) assay. The PCR products (~647 bp) were digested into two fragments (~465 and ~182 bp), indicative of effective CCR5 disruption. The non-transfection control is indicated as “Mock”.

Stripped membrane

Probed with anti-β-Actin antibody

Probed with anti-CCR5 antibody


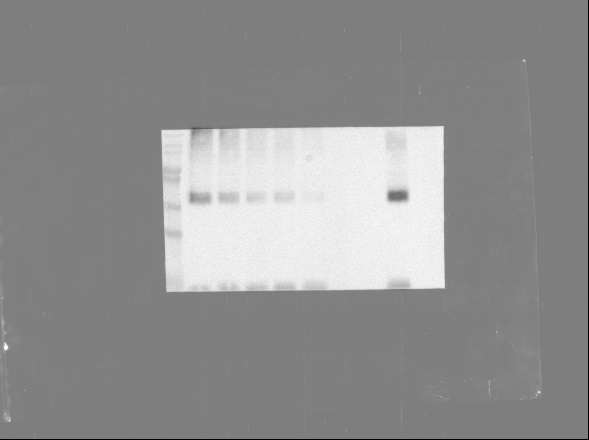

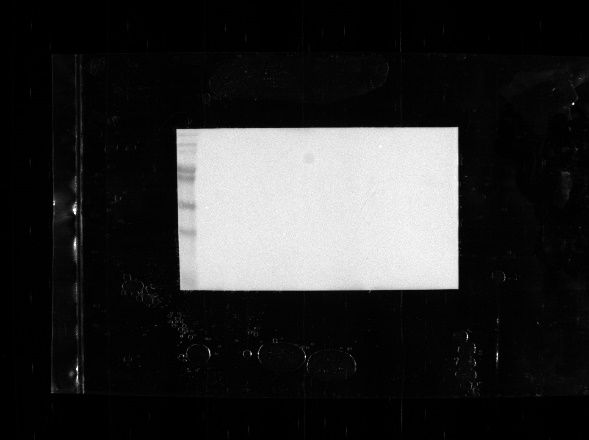

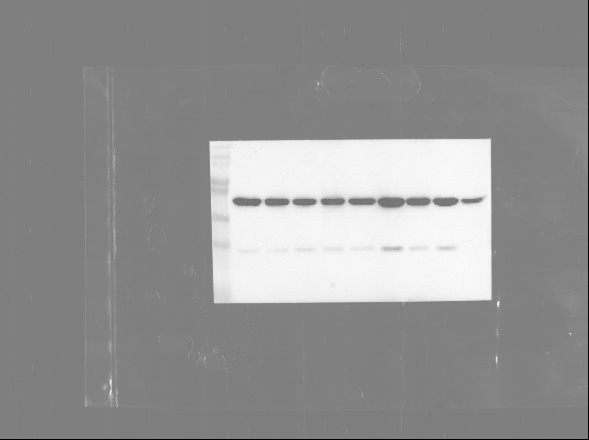


**Supplementary Information 2**

The original membrane photos of Fig. 1b illustrate the following: the top membrane was stained with anti-CCR5 antibody, the middle membrane represents the stripped membrane, and the lower membrane was probed with anti-β-Actin antibody.


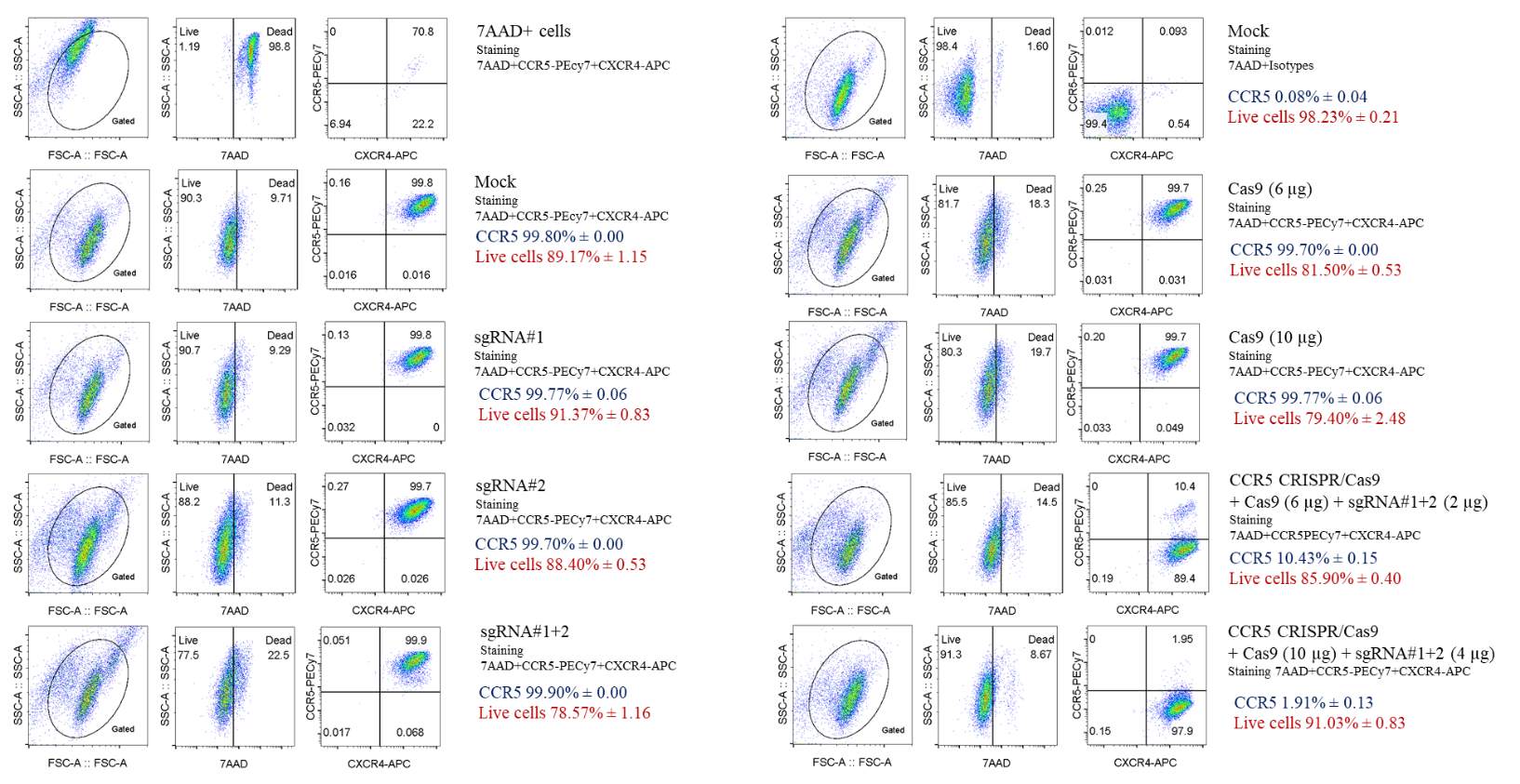


**Supplementary Information 3**

The flow cytometry analysis of CCR5 expression in MT4CCR5 cells was conducted three days post-transfection with individual sgRNA, the in-house Cas9 protein, or the combined sgRNAs with the in-house Cas9 protein. Representative flow cytometry plots demonstrate the gated cell population determined by forward scatter (FSC) and side scatter (SSC) in the left panel. Subsequently, only live cells, as identified by the absence of 7AAD staining (7AAD^-^ population), were further analyzed for CCR5 expression by staining with anti-CCR5 antibody conjugated with PECy7 (CCR5-PECy7). Additionally, the other HIV-1 co-receptor, CXCR4, stained with anti-CXCR4 antibody conjugated with APC (CXCR4-APC) was performed as a negative control for CCR5 reduction. The numbers indicated on the right side represent the gene expression levels as a percentage, with mean ± SD calculated from triplicate experiments.


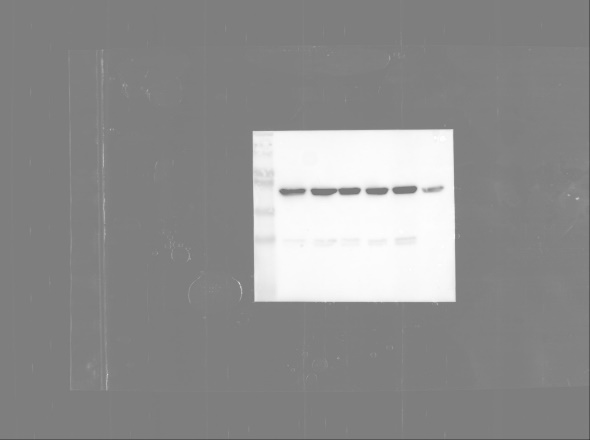

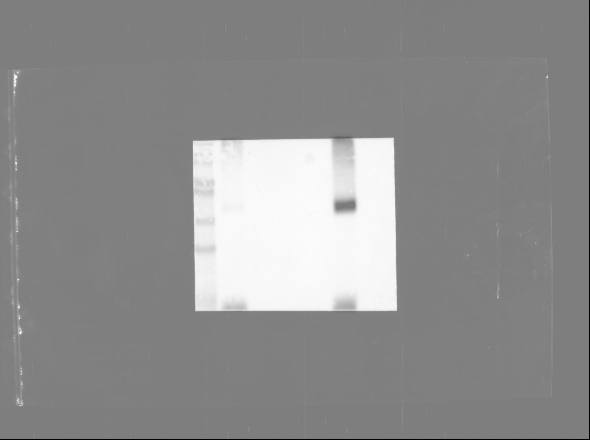

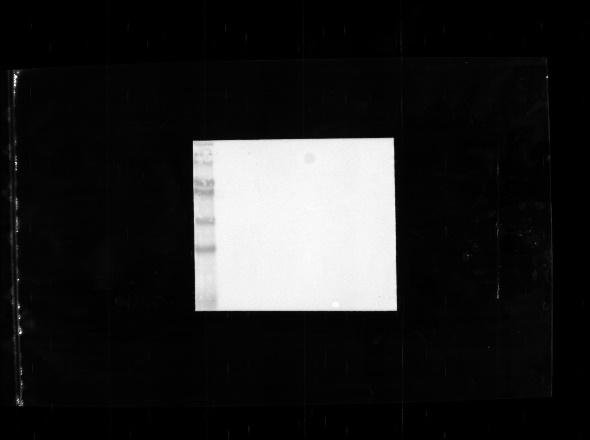


Probed with anti-β-Actin antibody

Stripped membrane

Probed with anti-CCR5 antibody

**Supplementary Information 4**

The original membrane photos of Fig. 3a illustrate the following: the top membrane was stained with anti-CCR5 antibody, the middle membrane represents the stripped membrane, and the lower membrane was probed with anti-β-Actin antibody.

**
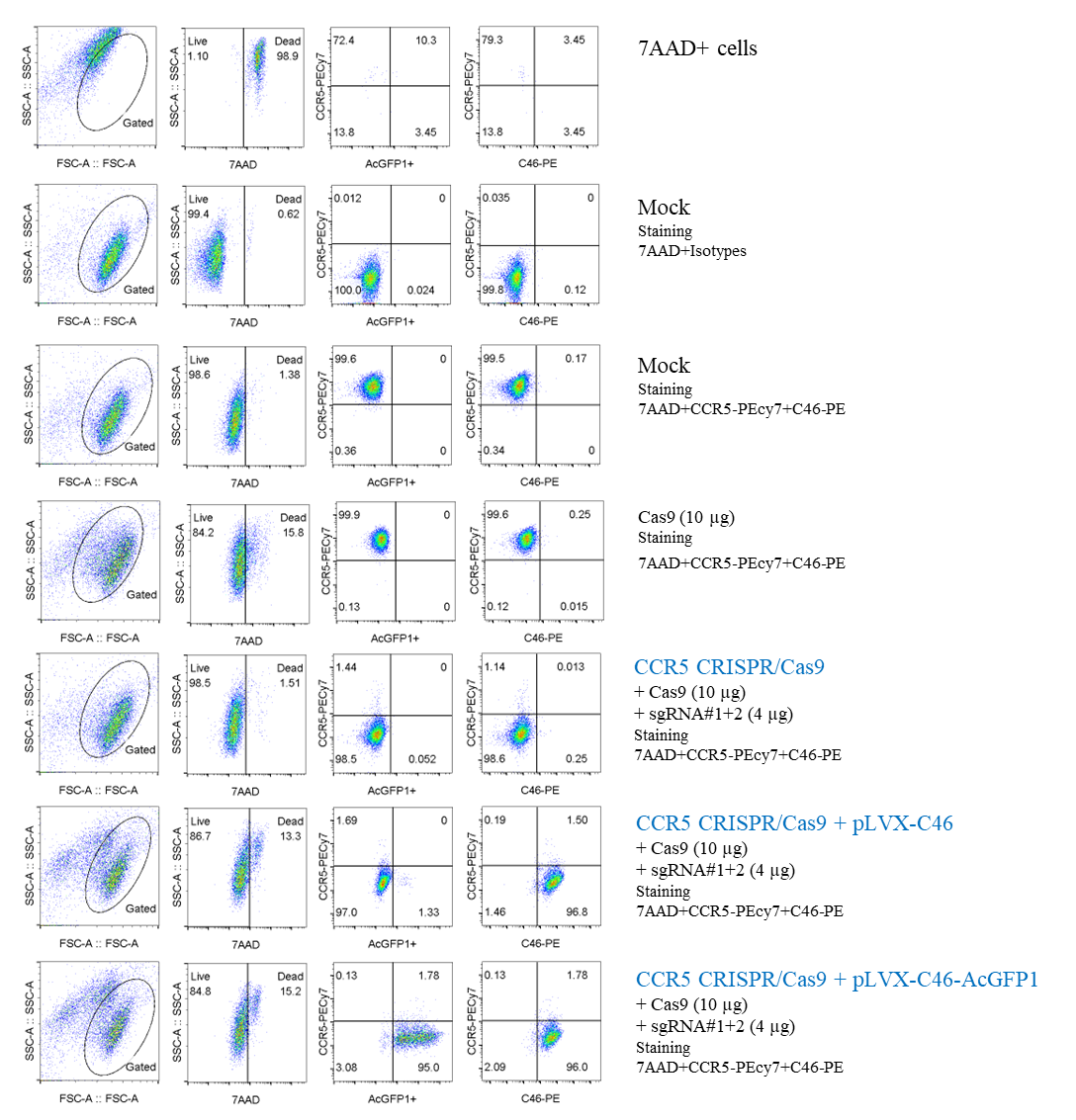
Supplementary Information 5**

The flow cytometry plots display representative data from the flow cytometry analysis of CCR5 reduction and the expression of the C46 HIV-1 fusion inhibitor on the cell membrane. Cells treated with the combined CRISPR/Cas9 knockout CCR5 and C46 HIV-1 fusion inhibitor, both with and without the AcGFP1 construct, as well as those transfected with single in-house Cas9 protein and mock control cells, were stained for the analysis of CCR5 expression using anti-CCR5 antibody conjugated with PECy7 (CCR5-PECy7). The expression of the C46 HIV-1 fusion inhibitor was determined by either the presence of AcGFP1+ or using the anti-2F5 antibody specific to the C46 peptide, followed by staining with anti-human IgG conjugated with PE (C46-PE).

**
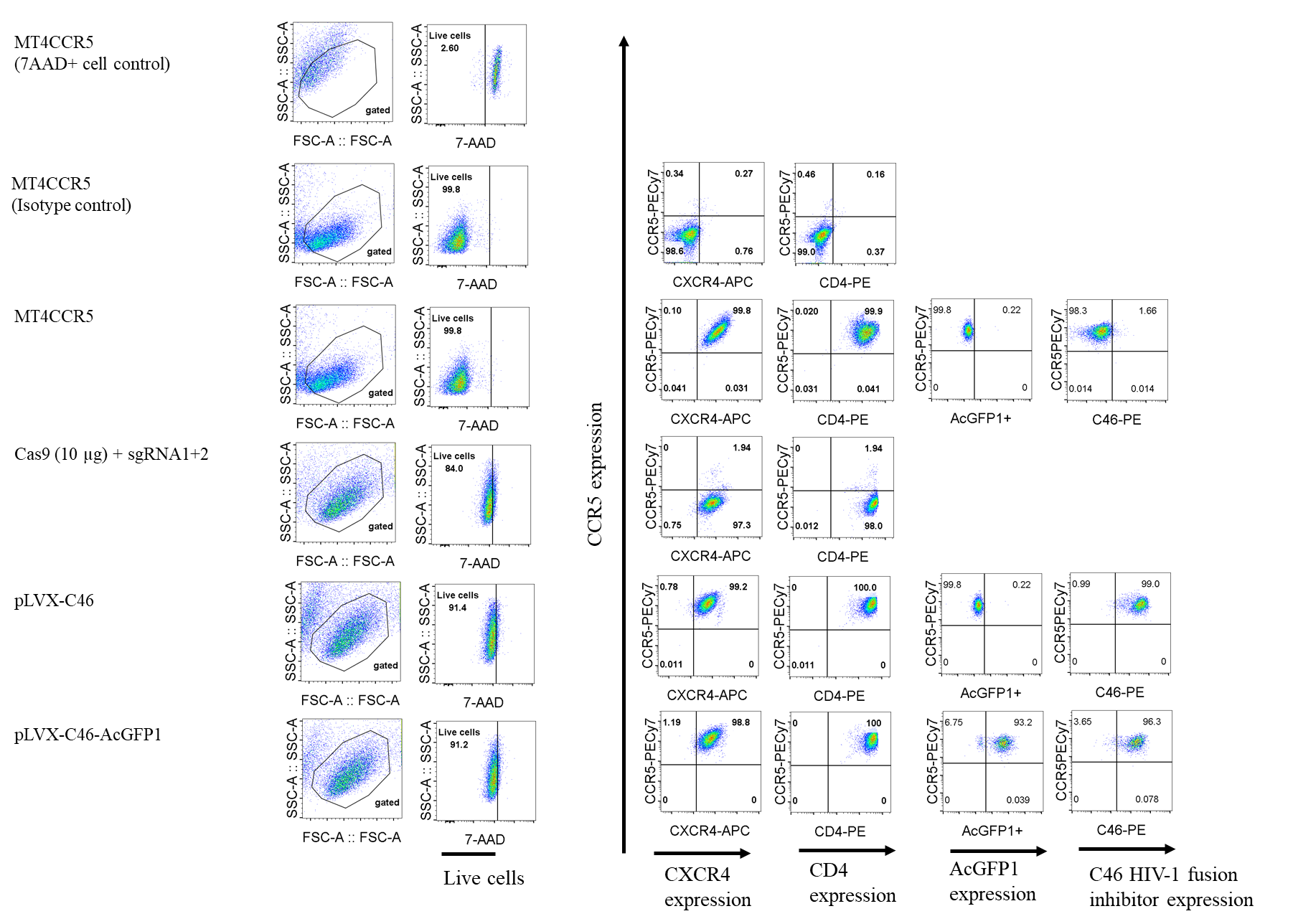
**

**Supplementary Information 6**

MT4CCR5 cells, treated with CRISPR/Cas9 knockout CCR5, pLVX-C46, and pLVX-C46-AcGFP1, along with mock cells, were assessed for the expression levels of CCR5, CXCR4, and CD4 using flow cytometry. The population of live cells, assessed via 7AAD^-^ staining, was further analyzed for expression levels of the HIV-1 receptor, HIV-1 co-receptor, and C46 expression levels. CCR5 expression was determined by anti-CCR5 antibody conjugated with PECy7 (CCR5-PECy7), while CXCR4 expression was evaluated by anti-CXCR4 antibody conjugated with APC (CXCR4-APC). CD4 expression was measured using anti-CD4 antibody conjugated with PE (CD4-PE). The cells transduced with pLVX-C46 or pLVX-C46-AcGFP1 were verified C46 expression levels by separating staining with anti-HIV-1 gp41 (C46-PE).

**
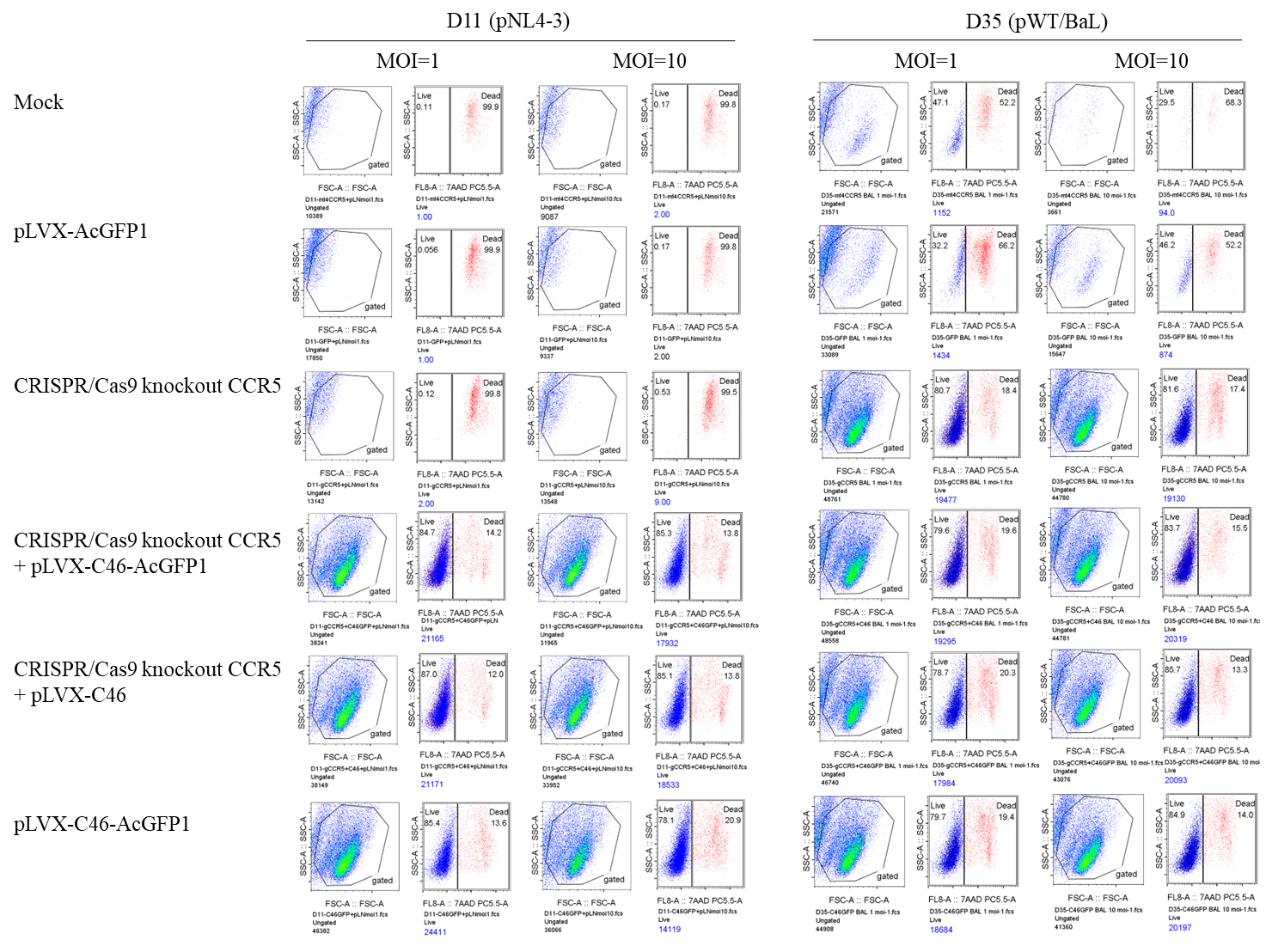
**

**Supplementary Information 7**

The number of live cells post-HIV-1 infection was determined by flow cytometry. Infected cells were stained with 7AAD, whereby cell death was verified by 7AAD^+^ cell population, while live cells were identified by the 7AAD^-^ cell population. The blue numbers indicate the quantity of live cells.
